# In response to: PSMA PET/CT cost-effectiveness analysis in the USA: a response to a published commentary

**DOI:** 10.1007/s00259-023-06398-8

**Published:** 2023-08-18

**Authors:** Adrien Holzgreve, Wolfgang G. Kunz, Dirk Mehrens, Marcus Unterrainer

**Affiliations:** 1 Department of Nuclear Medicine, LMU University Hospital, LMU Munich, Marchioninistr, 15, 81377 Munich, Germany; 2 Department of Radiology, University Hospital, LMU Munich, Munich, Germany

Dear Prof. Chiti, dear Editor-in-Chief,

We read with interest a published letter that reflects on our short communication entitled “Is PSMA PET/CT cost-effective for the primary staging in prostate cancer? First results for European countries and the USA based on the proPSMA trial”, recently published in the European Journal of Nuclear Medicine and Molecular Imaging [1, 2]. We thank the authors for the exchange taking place regarding this interesting topic. However, we perceive that statements in our paper might have led to subjective misunderstandings and would be happy to help clarify them in this reply.

Effectiveness can be measured with different parameters based on data availability, which pre-mandates the options for long-term modelling in cost-effectiveness analyses. Throughout the manuscript, we did not claim that a comprehensive cost-effectiveness analysis was performed. A “true cost-effectiveness analysis” was neither intended nor did we claim to have performed one. On the contrary, we explicitly state in the manuscript “Of note, the performed analysis is not a classical cost-effectiveness analysis trying to take into account all subsequent decision points and downstream costs based on outcomes, as there is not yet the level of evidence for modelling long-term effects. Therefore, in analogy to the Australian cost-effectiveness study, the cost per accurate diagnosis was chosen as the endpoint of our analysis”.

Further, the letter insinuates that “The authors state that their analysis is the first cost-effectiveness study for PSMA PET/CT performed in the US setting”. This is not true as well. In fact, we wrote: “This is the first study to analyse cost-effectiveness of PSMA PET/CT compared to conventional imaging based on results of the proPSMA trial for European and the US-American settings”. We would like to point out that this sentence is correct. Our analysis was based completely on data of the proPSMA trial, as described in the manuscript. We clearly referred to the proPSMA trial both in the title and in the sentence quoted above. Also, our analysis included European countries and the USA, as stated both in the title and in the sentence quoted above.

Moreover, the authors seek acknowledgement for their published work “as the first cost-effectiveness study performed in the USA for PSMA PET/CT”, referring to their article “Complex implementation factors demonstrated when evaluating cost-effectiveness and monitoring racial disparities associated with [^18^F] DCFPyL PET/CT in prostate cancer men”, published in “Scientific Reports” shortly after the submission of our short communication to the European Journal of Nuclear Medicine and Molecular Imaging. Even if the authors’ paper may have been the first paper published dealing with this topic in the USA, irrespective of our published article, this does not constitute the absolute need of citing this work as a consequence. As mentioned in the manuscript and above, our analysis focused on the data from the proPSMA trial only, so that no content-wise link to this manuscript is present.

In the light of the demanded literal accuracy, we wanted to point out that our previously published article in the EJNMMI was not classified as “commentary” but “short communication”.

Again, we thank the authors of the letter for their comments and consider it fortunate that the issue of cost-effectiveness of PSMA PET/CT is receiving the deserved attention taking place in the EJNMMI. We are looking forward to further exchanging scientifically on this topic.

## Data Availability

Not applicable
